# Drug-coated balloon, drug-eluting stent, versus bare-metal stent for vertebral artery ostial stenosis: a systematic review and network meta-analysis

**DOI:** 10.3389/fneur.2026.1901781

**Published:** 2026-10-08

**Authors:** Zhiqiang Li, Shuhui Wu, Chao Wu, Yue Yin, Xiaofan Xu

**Affiliations:** 1 Department of Neurology, Liaocheng People's Hospital, Liaocheng, China; 2 Department of Traditional Chinese Medicine, The Third People's Hospital of Liaocheng, Liaocheng, China; 3 Department of Neurology, Liaocheng Third People’s Hospital, Liaocheng, Shandong, China

**Keywords:** bare-metal stent, drug-coated balloon, drug-eluting stent, endovascular treatment, network meta-analysis, restenosis, vertebral artery ostial stenosis

## Abstract

**Background:**

Stenosis at the origin of the vertebral artery (VAOS) accounts for approximately one in five posterior circulation ischemic events. Bare-metal stents (BMS) have been the conventional endovascular treatment, but restenosis occurs in 11–67% of cases. Drug-coated balloons (DCB) and drug-eluting stents (DES) are alternative strategies; however, no network meta-analysis has compared all three modalities specifically at the vertebral origin.

**Methods:**

Five databases—PubMed, Embase, Cochrane CENTRAL, CNKI, and Wanfang—were searched from 2010 through May 2026 for studies comparing at least two of DCB, DES, or BMS in adults with symptomatic VAOS. The search identified 499 records; 393 underwent title and abstract screening after deduplication, from which 60 full-text articles were assessed. A frequentist random-effects network meta-analysis was conducted using the netmeta package (R 4.5.2), with Bayesian sensitivity analysis. The primary endpoint was imaging-confirmed restenosis (≥50%). The protocol was registered with PROSPERO (CRD420261413981).

**Results:**

Seven studies (three randomized trials and four observational cohorts, *N* = 970; range 46–217) met the inclusion criteria. Compared with BMS, both DES (OR 0.27, 95% CI 0.17–0.45) and DCB (OR 0.39, 95% CI 0.18–0.83) reduced restenosis. DES and DCB did not differ significantly from each other (OR 1.42, 95% CI 0.58–3.50). Between-study heterogeneity was modest (*I*^2^ = 20.5%). Although the point estimate suggests modest heterogeneity, the wide confidence interval and the small number of studies limit precision. Low *I*^2^ in a small network should not be equated with clinical homogeneity. P-scores ranked DES first (88.9%), DCB second (60.7%), and BMS last (0.4%). For perioperative safety, DCB ranked highest (P-score 90.0%).

**Conclusion:**

Both DES and DCB reduce restenosis compared with BMS in VAOS. Both DES and DCB are associated with lower restenosis rates than BMS. The relative anti-restenosis efficacy of DES versus DCB remains uncertain due to the absence of direct head-to-head comparisons. Treatment selection should be individualized based on lesion characteristics and procedural considerations. The DCB evidence base remains small, and head-to-head comparisons of DES versus DCB at the vertebral origin are needed.

**Systematic review registration:**

https://www.crd.york.ac.uk/PROSPERO/view/CRD420261413981, Identifier: CRD420261413981.

## Introduction

Vertebral artery ostial stenosis (VAOS), atherosclerotic narrowing of the V1 segment, is responsible for roughly 20% of posterior circulation ischemic events (1, 2). Among patients whose symptoms persist despite medical therapy, the annual risk of recurrent stroke or death reaches 5–11% (3). Current guidelines recommend intensive pharmacotherapy as first-line management, reserving endovascular revascularization for patients with refractory symptoms (4, 5). The 2023 European Society for Vascular Surgery (ESVS) Clinical Practice Guidelines similarly recommend intensive pharmacotherapy as first-line management for VAOS, with endovascular intervention reserved for patients with recurrent symptoms despite optimal medical therapy (6).

BMS implantation has been the most commonly used interventional approach, with high rates of acute technical success (7). However, neointimal hyperplasia drives restenosis in 11–67% of treated segments (8). DCB deliver antiproliferative agents without leaving a permanent scaffold, theoretically reducing restenosis while avoiding implant-related complications (9). DES combine mechanical scaffolding with sustained local drug release and have outperformed BMS in coronary and peripheral vascular beds (10).

Several individual studies have compared DCB against BMS (11, 12) or DES against BMS (13–16) in VAOS, with generally favorable but somewhat variable results. Two pairwise meta-analyses pooled DCB-versus-BMS comparisons (17, 18) —one of which (18) was conducted by our group and limited to single-arm DCB studies—and two others addressed DES versus BMS (19, 20); each reported advantages for the drug-based modality. A 2026 network meta-analysis by Xu et al. (21) combined all three modalities but included both intracranial and extracranial stenoses, introducing clinical heterogeneity (*I*^2^ up to 35%) that weakens site-specific inferences. Recently, large clinical series by Hajiyev et al. have provided contemporary real-world evidence on VAOS endovascular treatment patterns and restenosis management (22, 23). The present NMA was designed to close this gap by restricting eligibility to VAOS, thereby providing lesion-specific comparative estimates for all three strategies within a unified framework.

## Methods

### Protocol and registration

This review followed the PRISMA-NMA guidelines (24) and was prospectively registered with PROSPERO (CRD420261413981).

### Search strategy

We searched PubMed, Embase, Cochrane CENTRAL, CNKI, and Wanfang from January 2010 through May 2026. Complete search strategies appear in Supplementary Table S1. In brief, terms for vertebral artery stenosis, the V1 segment or ostium, and endovascular interventions (DCB, DES, BMS, and related variants) were combined. No language restrictions were applied.

### Eligibility criteria

Studies were eligible if they (a) enrolled adults (≥18 years) with symptomatic atherosclerotic VAOS, (b) compared at least two of DCB, DES, or BMS, and (c) reported at least one outcome of interest (imaging-confirmed restenosis, target-lesion revascularization [TLR], or perioperative complications). We excluded single-arm studies, case reports, reviews, studies restricted to restenosis, and studies in which the stenosis site could not be confirmed as the vertebral origin or V1 segment.

### Study selection and data extraction

Two reviewers independently screened titles and abstracts, then examined full-text articles against eligibility criteria. Disagreements were resolved through discussion with a third reviewer. The same two reviewers independently extracted data using a standardized form: study characteristics, patient demographics, lesion features, intervention details, and outcome events. The PRISMA flow of study selection appears in Supplementary Figure S1. Device-level details, including platform, drug coating and manufacturer, are listed in Supplementary Table S5.

### Outcomes

The primary outcome was imaging-confirmed restenosis (≥50%) at the latest reported follow-up. Secondary outcomes were TLR and perioperative complications (stroke, TIA, or death within 30 days).

### Risk of bias assessment

Randomized trials were assessed with the Cochrane Risk of Bias 2.0 tool; observational studies were evaluated with the Newcastle–Ottawa Scale (NOS). Two reviewers performed assessments independently, with disagreements resolved by consensus. Results are shown in Supplementary Figures S2, S3 (panels A and B, respectively). Item-level risk-of-bias judgements and total NOS scores for every included study are given in Supplementary Table S4.

### Statistical analysis

A frequentist random-effects network meta-analysis was conducted using the netmeta package (version 3.5–0) in R 4.5.2 (25). Odds ratios with 95% confidence intervals were estimated for binary outcomes. Between-study heterogeneity was quantified with *τ*^2^ and *I*^2^. Treatment rankings were derived from P-scores, which estimate the probability that a treatment is the best option (26). Node-splitting assessed local inconsistency between direct and indirect evidence (27). A Bayesian random-effects NMA with non-informative priors served as a sensitivity analysis. Comparison-adjusted funnel plots were inspected for small-study effects (28). Leave-one-out analysis tested the influence of individual studies.

## Results

### Study selection and characteristics

Database searches yielded 499 records; 106 duplicates were removed. Of 393 records screened by title and abstract, 333 were excluded. Sixty full-text articles were assessed, and 53 were excluded (14 lacked a DCB/DES/BMS comparator, 35 had inadequate outcome data, 2 represented overlapping cohorts, and 2 were unavailable). Seven studies met the inclusion criteria (Supplementary Figure S1).

The seven included studies—three RCTs (11–13) and four observational cohort studies (14–16, 29)—enrolled 970 patients overall (range 46–217; Table 1). Five studies contributed direct DES-versus-BMS comparisons (*n* = 759), three contributed DCB-versus-BMS comparisons (*n* = 274), and one small three-arm study contributed a DCB-versus-DES-versus-BMS comparison (*n* = 46). Mean ages ranged from 64 to 68 years; the proportion of men ranged from 64 to 84%. The angiographic stenosis threshold for enrollment was ≥70% in four studies and ≥50% in three. Mean follow-up was 12–16 months. Notable clinical differences across studies included variation in the angiographic stenosis threshold for enrollment (50% in three studies versus 70% in four), differences in DAPT duration (1–6 months), use of different DES platforms (sirolimus-eluting, everolimus-eluting, and paclitaxel-eluting), and the predominance of male participants (64–84%).

**Table 1:** Characteristics of included studies.

Study	Design	Total *N*	DCB	DES	BMS	Mean Age	Male%	Stenosis	FU (mo)	Outcomes
Luo 2026 (11)	RCT	179	91	–	88	64	72%	> = 70%	12	Restenosis, TLR, Periop.
Wang 2021 (12)	RCT	95	49	–	46	67	75%	> = 70%	16	Restenosis, TLR, Periop.
He 2019 (13)	RCT	132	–	66	66	64	68%	> = 50%	12	Restenosis, TLR, Periop.
Li 2020 (14)	Cohort	110	–	55	55	65	64%	> = 50%	14	Restenosis, TLR
Wang 2022 (15)	Cohort	46	5	29	12	67	71%	> = 70%	14	Restenosis, TLR, Periop.
Sun 2025 (29)	Cohort	217	–	150	67	NR	NR	> = 50%	12	Restenosis, Periop.
Zhao 2026 (16)	Cohort	191	–	93	98	68	84%	> = 70%	12	Restenosis, TLR, Periop.

DCB, drug-coated balloon; DES, drug-eluting stent; BMS, bare-metal stent; FU, follow-up; NR, not reported; Periop., perioperative complications; RCT, randomized controlled trial; TLR, target-lesion revascularization.

### Risk of bias

Two of the three RCTs (11, 12) were judged to have some concerns, driven by missing outcome data (Supplementary Figure S2A/B, Supplementary Table S4). The four cohort studies scored 8–9 out of 9 on the NOS (Supplementary Figure S3A/B); the main limitation was the retrospective design of three of them.

### Network meta-analysis: restenosis

The network geometry is shown in Figure 1. Compared with BMS, restenosis was significantly lower with both DES (OR 0.27, 95% CI 0.17–0.45, *p* < 0.001) and DCB (OR 0.39, 95% CI 0.18–0.83, *p* = 0.015; Figure 2). DES and DCB did not differ significantly (OR 1.42, 95% CI 0.58–3.50, *p* = 0.440; Table 2). Between-study heterogeneity was modest (*τ*^2^ = 0.078; *I*^2^ = 20.5, 95% CI 0.0–64.0%). P-scores ranked DES first (88.9%), DCB second (60.7%), and BMS last (0.4%; Table 2; Figure 3). Based on the pooled event rates from the NMA model, the estimated absolute risk of restenosis was 26.9% for BMS, 12.5% for DCB, and 9.1% for DES (model-predicted from NMA). Compared with BMS, DCB was associated with an absolute risk reduction (ARR) of 14.3 percentage points (95% CI 3.5–20.6; NNT 7, 95% CI 5–29), and DES with an ARR of 17.7 percentage points (95% CI 12.6–21.1; NNT 6, 95% CI 5–8).

**Figure 1:**
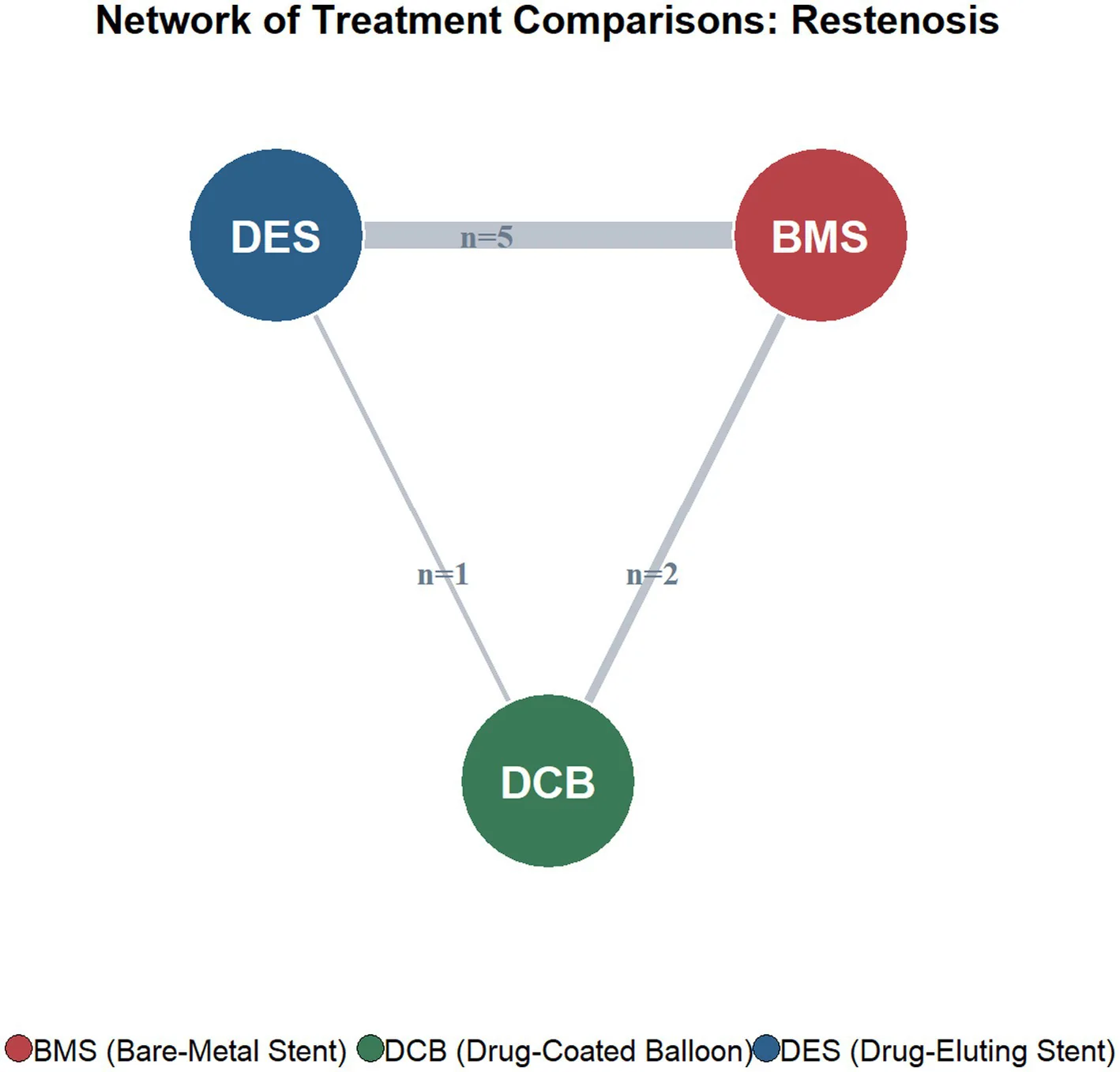
Network geometry. Node size is proportional to the number of patients per treatment; edge thickness is proportional to the number of studies per comparison. BMS, bare-metal stent; DCB, drug-coated balloon; DES, drug-eluting stent.

**Figure 2:**
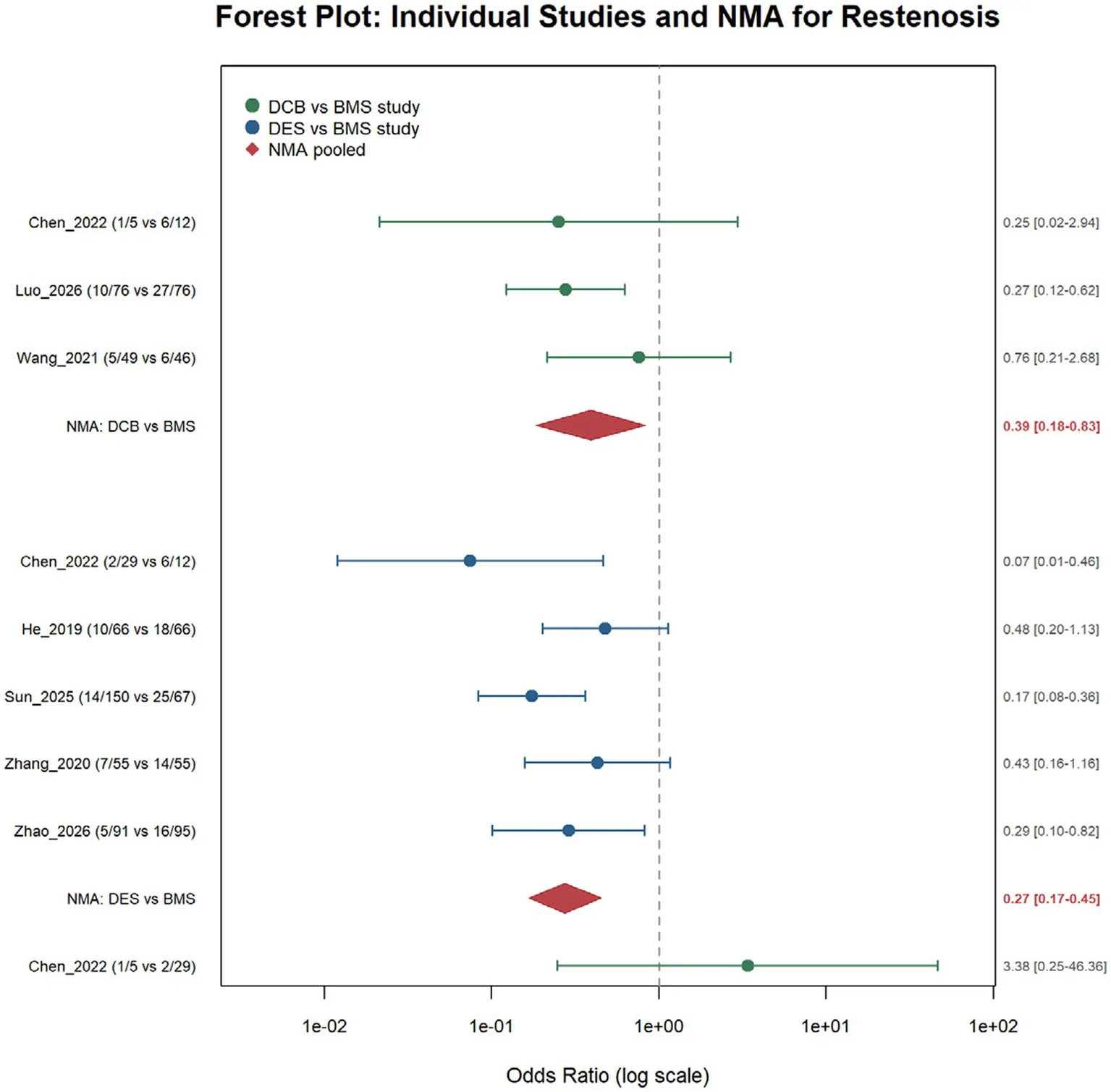
Forest plot of network meta-analysis results for restenosis (bare-metal stent as reference). CI, confidence interval; OR, odds ratio; BMS, bare-metal stent; DCB, drug-coated balloon; DES, drug-eluting stent.

**Table 2:** Network meta-analysis results for restenosis.

Comparison	NMA OR (95% CI)	*p*-value	Direct OR (95% CI)	*k*	*I* ^2^	P-score
DCB vs. BMS	0.39 (0.18–0.83)	0.015	0.37 (0.17–0.80)	3	0%	60.7%
DES vs. BMS	0.27 (0.17–0.45)	<0.001	0.27 (0.16–0.46)	5	30.9%	88.9%
DES vs. DCB	1.42 (0.58–3.50)	0.440	0.30 (0.02–4.81)	1	–	–

NMA, network meta-analysis; OR, odds ratio; CI, confidence interval; *k*, number of studies. *τ*^2^ = 0.078; *I*^2^ = 20.5% (95% CI 0.0–64.0%).

**Figure 3:**
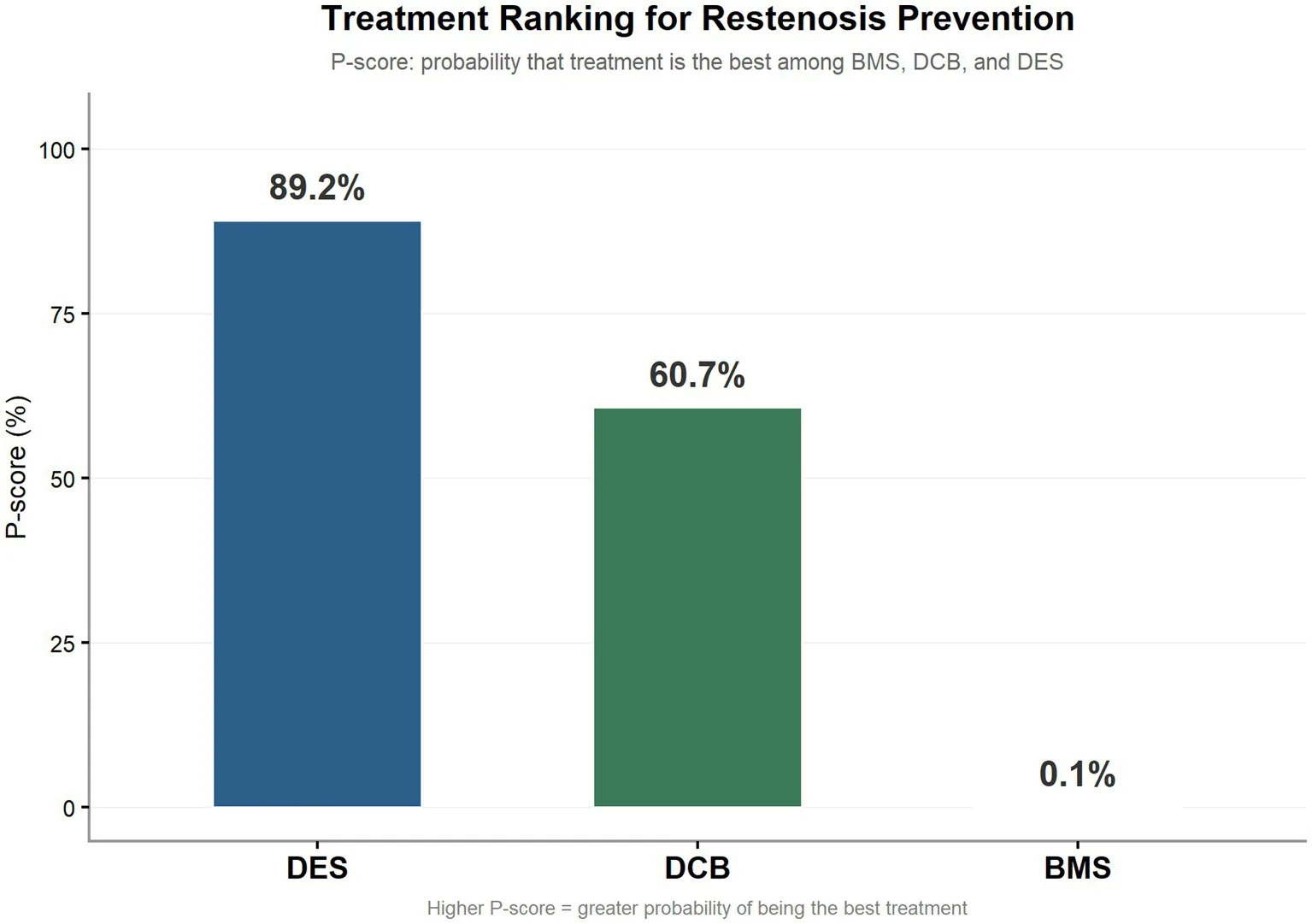
P-score ranking for restenosis. Higher P-score indicates higher probability of being the optimal treatment. BMS, bare-metal stent; DCB, drug-coated balloon; DES, drug-eluting stent.

### Inconsistency and sensitivity analyses

Node-splitting did not detect significant local inconsistency for any comparison (all *p* > 0.05; Figure 4). Leave-one-out analysis confirmed that no single study unduly influenced the primary findings (Figure 5). Bayesian sensitivity analysis produced results consistent with the frequentist model. The comparison-adjusted funnel plot showed no clear evidence of small-study effects (Figure 6).

**Figure 4:**
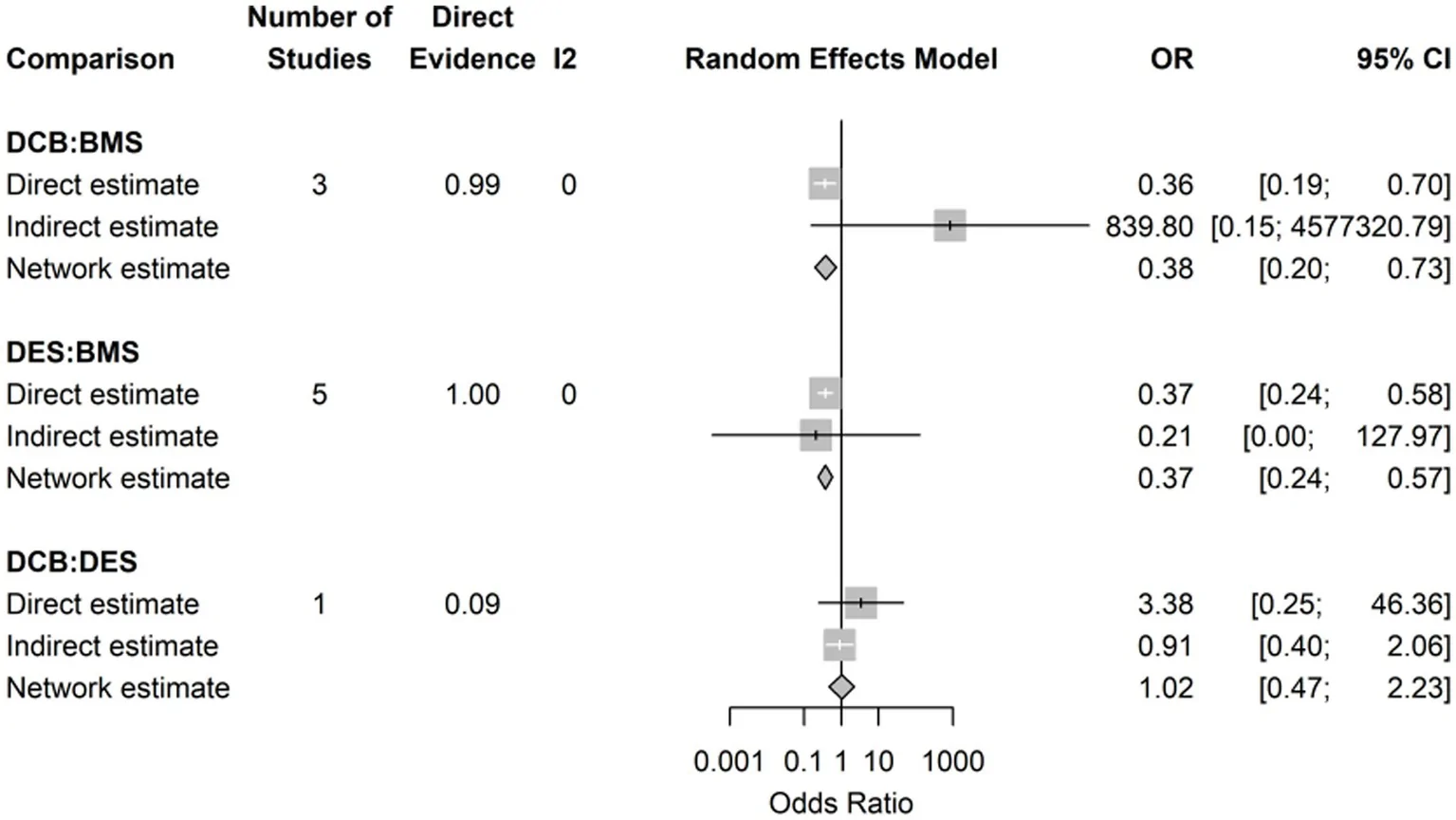
Node-splitting analysis. Comparison of direct and indirect evidence; *p* > 0.05 indicates no significant inconsistency. CI, confidence interval; BMS, bare-metal stent; DCB, drug-coated balloon; DES, drug-eluting stent.

**Figure 5:**
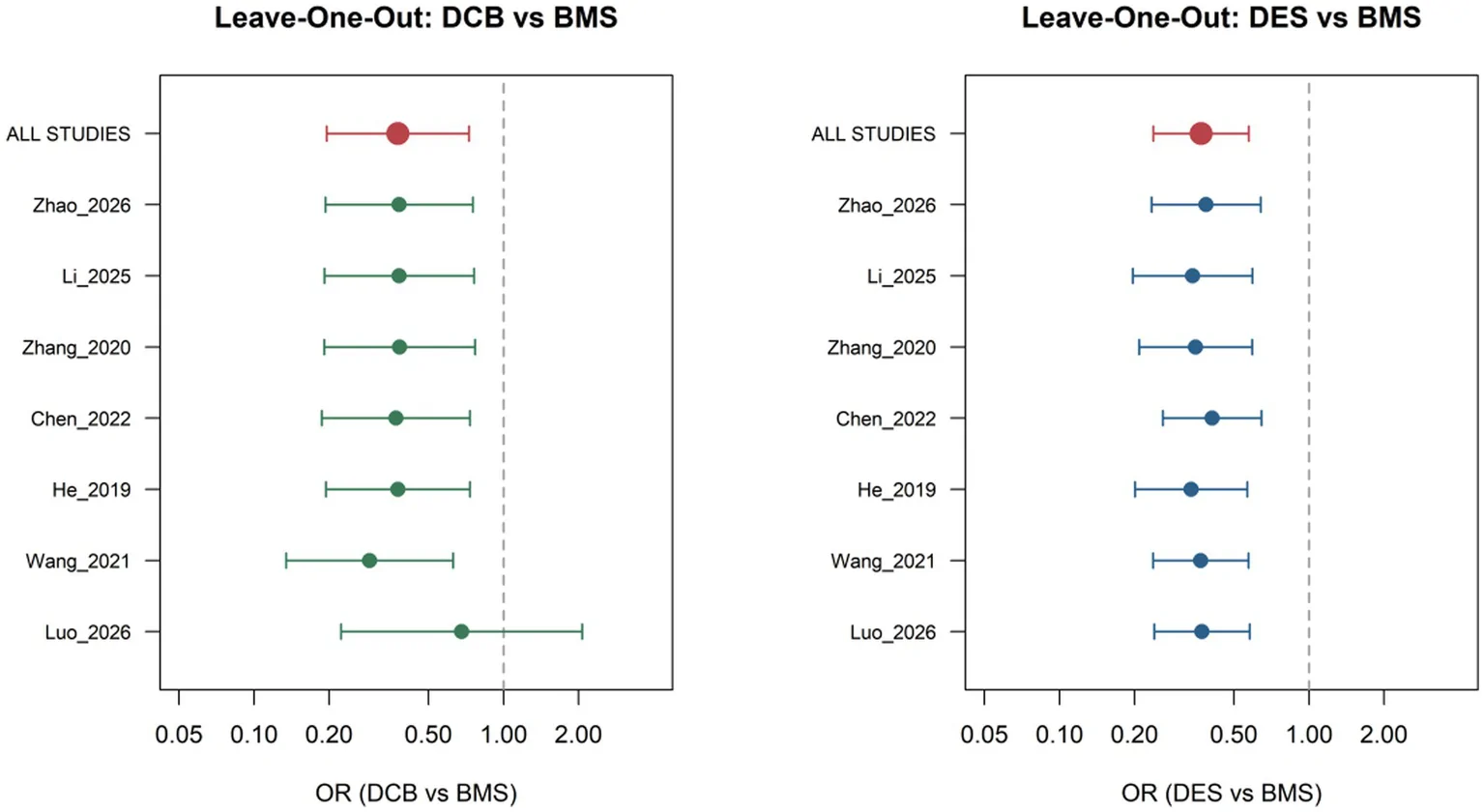
Leave-one-out sensitivity analysis. Each row shows the pooled estimate after omitting the corresponding study. CI, confidence interval; OR, odds ratio; BMS, bare-metal stent; DCB, drug-coated balloon.

**Figure 6:**
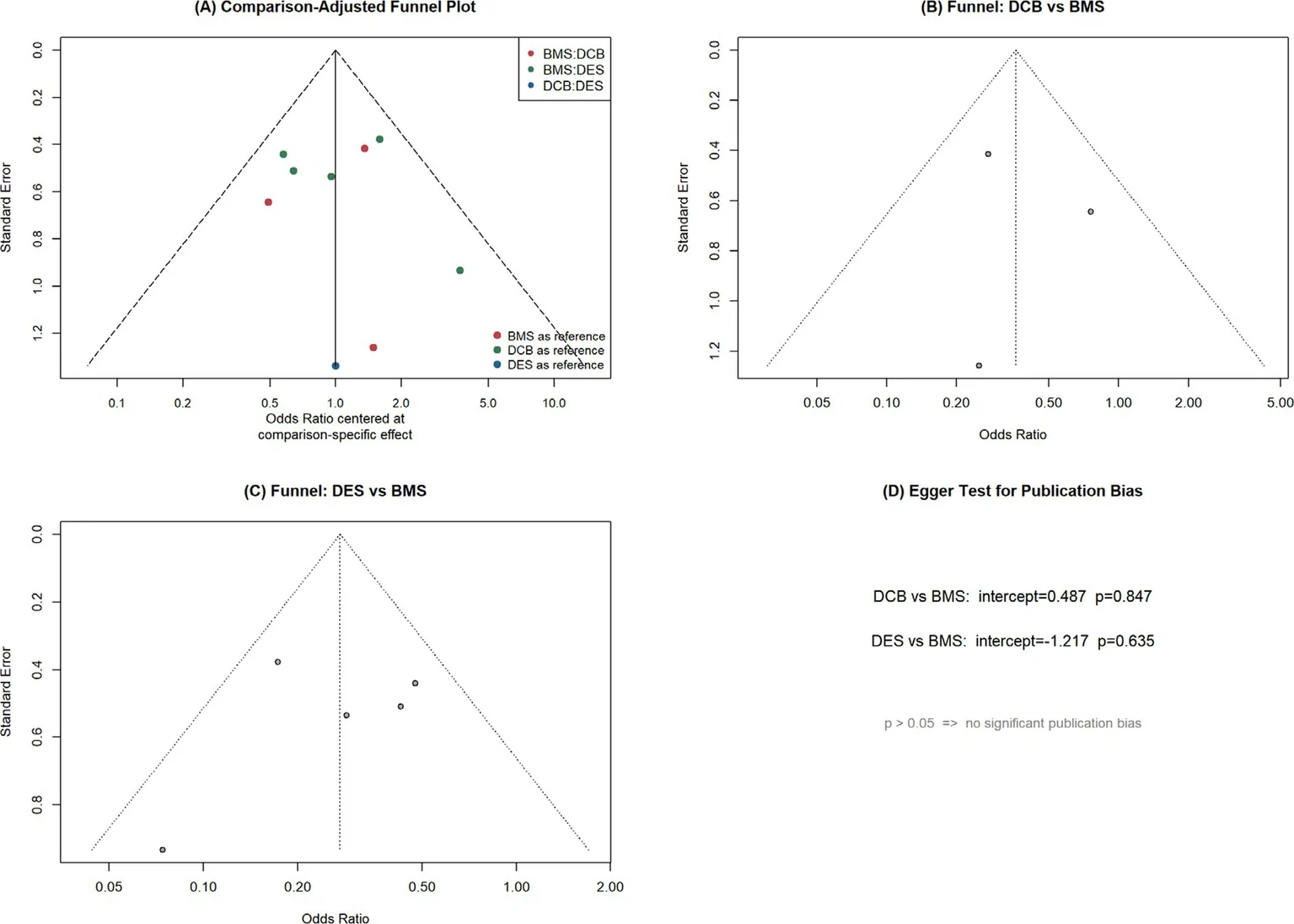
**(A)** Comparison-adjusted funnel plot; **(B)** funnel plot for DCB versus BMS; **(C)** funnel plot for DES versus BMS; **(D)** Egger’s test results. Symmetry suggests the absence of small-study effects and publication bias. SE, standard error; BMS, bare-metal stent; DCB, drug-coated balloon; DES, drug-eluting stent.

### Secondary outcomes

For TLR, P-scores ranked DES highest (76.2%), followed by DCB (63.4%) and BMS (10.4%; Table 2). For perioperative complications, DCB ranked highest (90.0%), followed by DES (55.0%) and BMS (5.0%). The individual components of perioperative complications (stroke, TIA, and death) are reported by treatment arm in Supplementary Table S2. A league-table heatmap summarizing all outcomes appears in Figure 7.

**Figure 7:**
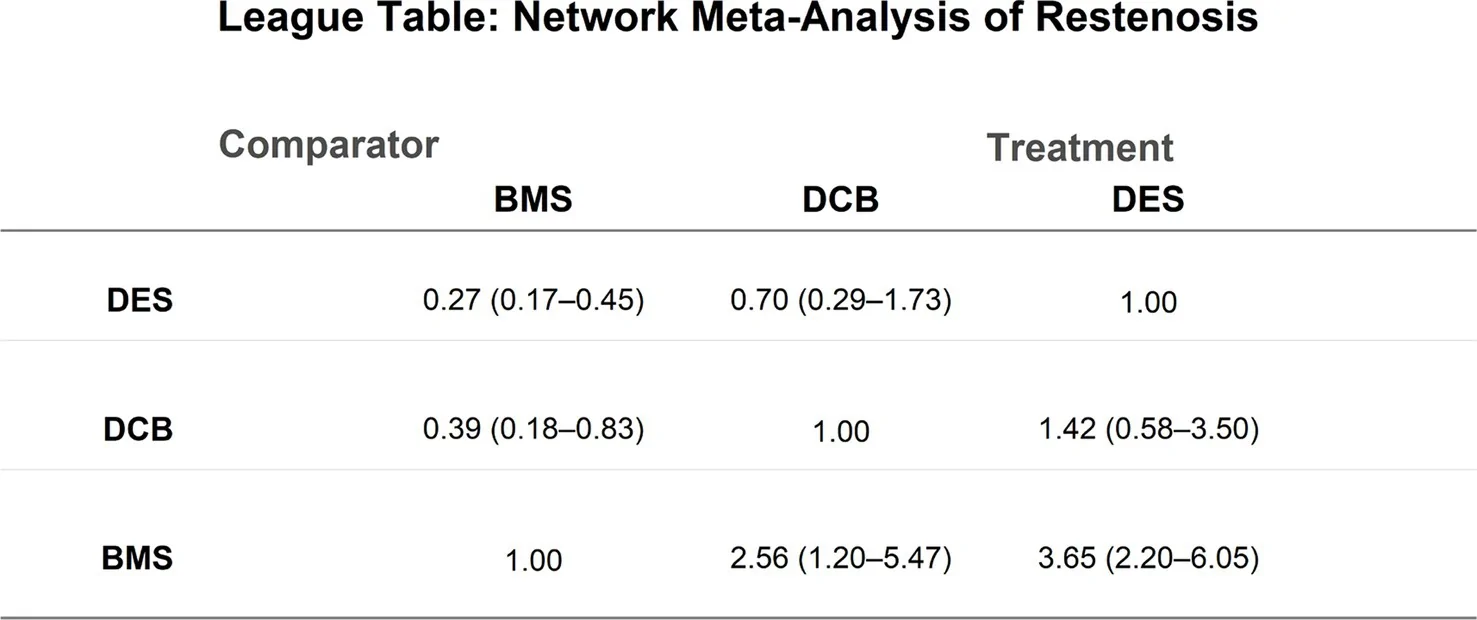
League table of network meta-analysis and direct-comparison odds ratios. Lower triangle: Network meta-analysis odds ratios. Upper triangle: Direct-comparison odds ratios. BMS, bare-metal stent; DCB, drug-coated balloon; DES, drug-eluting stent.

## Discussion

This network meta-analysis, restricted to studies of vertebral artery ostial stenosis, confirms that both DES and DCB substantially reduce restenosis compared with BMS. It extends earlier pairwise meta-analyses (17–19) —including an earlier report from our group that examined single-arm DCB data (18)—by simultaneously comparing all three modalities within a lesion-specific framework.

The absolute risk of restenosis after BMS exceeded 30% at one year, consistent with prior reports (8, 9). DES lowered the odds of restenosis by approximately 73% relative to BMS, and DCB by approximately 61%. Although the point estimates favor DES for restenosis prevention and DCB for procedural safety, the indirect DES-versus-DCB comparison should be interpreted with considerable caution. No direct head-to-head randomized trial of DES versus DCB for VAOS has been conducted. This comparison is dominated by one small three-arm study (Wang (15), *n* = 46), and the confidence interval is wide (OR 1.42, 95% CI 0.58–3.50), crossing the null. The P-score ranking (DES 88.9% versus DCB 60.7%) is driven predominantly by the larger volume and greater precision of DES-versus-BMS comparisons relative to DCB-versus-BMS comparisons, rather than by a demonstrated superiority of DES over DCB. DCB is represented by only 145 patients across three studies, whereas DES data derive from 393 patients across six studies. The ranking of DES above DCB therefore reflects stronger evidence rather than a definitive efficacy advantage:it is dominated by one small three-arm study (Wang (15), *n* = 46), and the confidence interval is wide (OR 1.42, 95% CI 0.58–3.50). DCB is represented by only 145 patients across three studies, whereas DES data derive from 393 patients across six studies. The ranking of DES above DCB therefore reflects stronger evidence rather than a definitive efficacy advantage.

The favorable safety profile of DCB (P-score 90.0%) is consistent with the mechanism of avoiding a permanent implant: without a metallic scaffold, the risks of stent thrombosis, chronic inflammation, and late stent fracture are absent (9, 30, 31). However, DCB-treated vessels may be susceptible to acute elastic recoil or dissection requiring bailout stenting, which occurred in 22% of cases in the largest DCB trial (11).

Clinical Heterogeneity Across Included Studies. Although our NMA was restricted to VAOS, residual heterogeneity persists: the included DES and DCB varied in platform design, polymer type, and antiproliferative agent; DAPT duration ranged from 1 to 6 months; and stenosis thresholds, follow-up duration, and restenosis ascertainment methods differed across studies. These factors are known to influence restenosis risk independently of device type, and our analysis cannot fully disentangle their contributions from the treatment effect.

Several additional limitations warrant consideration. First, only seven studies (970 patients) were included, and the DES-versus-DCB comparison relies on a single small three-arm study with few events. Second, four of seven studies were observational, introducing potential confounding by indication. Third, follow-up was limited to 12–16 months, precluding assessment of long-term patency. Fourth, the transitivity assumption underlying NMA may be challenged by the aforementioned between-study differences, and the limited number of studies precludes formal evaluation. Fifth, grey literature and trial registries were not systematically screened. CINeMA assessment rated the certainty of evidence as moderate for DCB/DES versus BMS and low for DES versus DCB (Supplementary Table S3). Future individual participant data meta-analyses would allow covariate-level adjustment and strengthen the evidence base.

These data support the use of either DES or DCB over BMS in symptomatic VAOS amenable to endovascular treatment. For lesions with favorable anatomy and lower procedural complexity, DCB may offer adequate patency with a better safety margin. For longer, more complex, or heavily calcified lesions, DES may provide more reliable long-term patency. Treatment choice should account for lesion length, vessel tortuosity, operator experience, and patient bleeding risk. Adequately powered head-to-head RCTs comparing DES and DCB at the vertebral origin are needed to move beyond the indirect comparisons on which current evidence depends.

## Data Availability

The original contributions presented in the study are included in the article/Supplementary material, further inquiries can be directed to the corresponding author/s.

## Supplementary material

SUPPLEMENTARY FIGURE S1 PRISMA 2020 flow diagram of study selection.

SUPPLEMENTARY FIGURE S2A Risk-of-bias summary (Cochrane RoB 2.0) for the randomized trials.

SUPPLEMENTARY FIGURE S2B Risk-of-bias traffic-light plot (RoB 2.0) for the randomized trials.

SUPPLEMENTARY FIGURE S3A Newcastle–Ottawa Scale summary for the cohort studies.

SUPPLEMENTARY FIGURE S3B Newcastle–Ottawa Scale traffic-light plot for the cohort studies.

SUPPLEMENTARY Table S1 Database search strategies.

SUPPLEMENTARY Table S2 Perioperative complications (30-day) by treatment arm.

SUPPLEMENTARY Table S3 CINeMA certainty of evidence assessment.

SUPPLEMENTARY Table S4 Risk of bias assessment.

SUPPLEMENTARY Table S5 Devices used in included studies.
