# Supplementary material for: Drug-coated balloon, drug-eluting stent, versus bare-metal stent for vertebral artery ostial stenosis: a systematic review and network meta-analysis
Source: Front Neurol. 2026 Oct 8;17:1901781. doi: 10.3389/fneur.2026.1901781 (PMC13686859; doi:10.3389/fneur.2026.1901781)
Supplement: Supplementary file 1 [file Data_sheet_2.pdf]

NIHR | National Institute for Health and Care Research

PROSPERO
International prospective register of systematic reviews

Drug-coated balloon, drug-eluting stent, versus bare-metal stent for vertebral artery origin stenosis: an updated network meta-analysis

Zhiqiang Li, shuhui wu

Citation

Zhiqiang Li, shuhui wu. Drug-coated balloon, drug-eluting stent, versus bare-metal stent for vertebral artery origin stenosis: an updated network meta-analysis. PROSPERO 2026 CRD420261413981. Available from https://www.crd.york.ac.uk/PROSPERO/view/CRD420261413981.

REVIEW TITLE AND BASIC DETAILS

Review title

Drug-coated balloon, drug-eluting stent, versus bare-metal stent for vertebral artery origin stenosis: an updated network meta-analysis

Condition or domain being studied

Ischemic Stroke; Vertebral Artery Stenosis; Atherosclerosis; Angioplasty

Vertebral artery origin stenosis/occlusion is a major cause of posterior circulation ischemia and stroke. Atherosclerotic stenosis at the vertebral artery ostium accounts for approximately 20% of posterior circulation ischemic events. The optimal revascularization strategy—whether endovascular stenting, angioplasty alone, or medical management—remains debated due to high restenosis rates and procedural risks.

Rationale for the review

Vertebral artery origin stenosis accounts for approximately 9–20% of posterior circulation ischemic strokes, yet the optimal management strategy remains controversial. Existing randomized trials are underpowered, and prior systematic reviews are either outdated or combine heterogeneous lesion locations. An updated, focused meta-analysis confined to ostial lesions is needed to provide more precise estimates of restenosis rates, periprocedural complications, and long-term clinical outcomes, thereby informing evidence-based decision-making.

Review objectives

In adult patients (⩾18 years) with vertebral artery origin stenosis (VAOS, V1 segment), what are the comparative efficacy and safety of drug-coated balloon angioplasty (DCB), drug-

eluting stent implantation (DES), and bare-metal stent implantation (BMS)?

Keywords

Network meta-analysis; Systematic review; Posterior circulation ischemia; Endovascular treatment; Restenosis; Comparative effectiveness

Country

China

ELIGIBILITY CRITERIA

Population

Included

Adults (⩾18 years) with symptomatic or severe (⩾70%) asymptomatic vertebral artery origin stenosis (V1 segment) confirmed by DSA, CTA, or MRA. Both sexes.

Excluded

Lesions beyond the V1 segment (V2–V4 or intracranial vertebral artery); non-atherosclerotic etiologies (dissection, vasculitis, moyamoya disease, radiation-induced stenosis); in-stent restenosis as the primary indication; combined anterior-posterior circulation procedures.

Intervention(s) or exposure(s)

Included

Bare Metal Stent; Drug Eluding Stent

Group 1: Drug-coated balloon angioplasty (paclitaxel- or sirolimus-coated), with or without adjunctive bailout stenting for flow-limiting dissection.

Group 2: Drug-eluting stent implantation (sirolimus-, paclitaxel-, rapamycin-, or everolimus-eluting), balloon-expandable or self-expanding.

Excluded

Stenting after failed DCB with no DCB-only arm to compare; vertebral artery stenting combined with carotid or intracranial stenting in same procedure; bioactive-coated or covered stents.

Comparator(s) or control(s)

Included

PICO tags selected: Bare Metal Stent

Bare-metal stent implantation using any uncoated stent platform (balloon-expandable or self-expanding), including dedicated vertebral stents and coronary/peripheral stents used off-label for the vertebral artery ostium.

Study design
Both randomized and nonrandomized study types will be included.

Included
Randomised controlled trials (RCTs) and comparative cohort studies (retrospective or prospective) with ⩾2 of DCB/DES/BMS, minimum 5 patients per treatment arm, and ⩾3 months follow-up.

Excluded
Single-arm studies, case reports/series (n < 5), reviews/editorials, conference abstracts without full-text, in-stent restenosis as primary condition.

Context
No geographical restriction. Given the high procedural volume of vertebral artery origin stenosis endovascular treatment in China, Chinese-language databases (CNKI) were specifically searched alongside English-language databases. Studies conducted in any country and healthcare setting are eligible.

TIMELINE OF THE REVIEW

Date of first submission to PROSPERO
03 June 2026

Review timeline
Review start date: 1 June 2026. Review end date: 15 July 2026.

Date of registration in PROSPERO
03 June 2026

AVAILABILITY OF FULL PROTOCOL

Availability of full protocol
A full protocol has not been written.

SEARCHING AND SCREENING

Search for unpublished studies
Only published studies will be sought.

Main sources that will be searched
The main sources to be searched are *CENTRAL - Cochrane Central Register of Controlled Trials*, *CNKI - China National Knowledge Infrastructure database*, *Embase*, *MEDLINE*, *PubMed* and *SCIE - Science Citation Index Expanded*.

Other sources that will be searched
Wanfang Data, SinoMed

Search language restrictions
There are no language restrictions.

Search date restrictions

Databases will be searched for articles published from 1 May 2010 and before by 3 June 2026.

Other methods of identifying studies

Other studies will be identified by: *contacting authors or experts* and *reference list checking (backward citation searching)*.

Link to search strategy

A full search strategy is available in the full protocol as described in the *Availability of full protocol* section

Selection process

Studies will be screened independently by at least two people (or person/machine combination) with a process to resolve differences.

Other relevant information about searching and screening

Initial search yielded 612 records across 7 databases. After automated deduplication in EndNote (299 unique records), two reviewers independently screened titles/abstracts. Twenty-four full-text articles were assessed against eligibility criteria by both reviewers, with disagreements resolved by consensus. Seven studies (3 RCTs + 4 cohorts, N=931) met final inclusion criteria. The PRISMA 2020 flow diagram documents the complete screening process.

DATA COLLECTION PROCESS

Data extraction from published articles and reports

Data will be extracted independently by at least two people (or person/machine combination) with a process to resolve differences.

Authors will be asked to provide any required data not available in published reports.

Study risk of bias or quality assessment

Risk of bias will be assessed using: *Cochrane RoB-2* and *Newcastle-Ottawa*

Data will be assessed independently by at least two people (or person/machine combination) with a process to resolve differences.

Additional information will be sought from study investigators if required information is unclear or unavailable in the study publications/reports.

Reporting bias assessment

Comparison-adjusted funnel plot for the primary outcome (restenosis). Visual inspection for asymmetry was performed to detect potential small-study effects and publication bias. Given the limited number of included studies (k=7), formal statistical tests for funnel plot asymmetry (e.g., Egger's test) were not conducted due to insufficient power

Certainty assessment

CINeMA (Confidence in Network Meta-Analysis; Salanti et al., PLoS Med 2014) will be used to evaluate the certainty of evidence for each treatment comparison in the network. CINeMA applies the GRADE framework adapted for NMA, assessing six domains: within-

study bias, reporting bias, indirectness, imprecision, heterogeneity, and incoherence. Judgments will be summarized as high, moderate, low, or very low confidence for each comparison on the primary outcome (restenosis).

OUTCOMES TO BE ANALYSED

Main outcomes

Primary Outcome

Imaging-confirmed restenosis (⩾50% luminal narrowing within the treated V1 segment), assessed by DSA, CTA, or Doppler ultrasound at the latest follow-up (⩾3 months post-procedure). Effect measure: odds ratio (OR) with 95% confidence interval.

Additional outcomes

Secondary Outcomes

Target lesion revascularization (TLR) — clinically driven repeat intervention of the treated vertebral artery origin; measured at latest follow-up; OR with 95% CI.

Perioperative complications — composite of stroke, TIA, or all-cause death within 30 days of the index procedure; OR with 95% CI.

Technical success — residual stenosis <30% with TICI grade 3 antegrade flow immediately post-procedure; OR with 95% CI.

Stroke recurrence — any new posterior circulation ischemic stroke or TIA during follow-up; OR with 95% CI.

All-cause mortality — death from any cause during follow-up; OR with 95% CI.

PLANNED DATA SYNTHESIS

Strategy for data synthesis

Random-effects frequentist network meta-analysis (graph-theoretical approach, R package netmeta v3.5-0) with DerSimonian-Laird estimator for between-study heterogeneity. Dichotomous outcomes will be combined as odds ratios (OR) with 95% confidence intervals. A sensitivity analysis using Bayesian NMA (normal approximation to log-odds, vague $N(0, 100^2)$ priors, 100,000 Monte Carlo simulations) will provide posterior ORs with 95% credible intervals and SUCRA rankings. Direct pairwise random-effects meta-analysis (DerSimonian-Laird) will supplement comparisons with ⩾2 studies.

CURRENT REVIEW STAGE

Stage of the review at this submission

Review stage	Started	Completed
Pilot work	✓	✓
Formal searching/study identification	✓	✓
Screening search results against inclusion criteria	✓	✓
Data extraction or receipt of IPD		
Risk of bias/quality assessment		
Data synthesis		

Review status
The review is currently planned or ongoing.

Publication of review results
Results of the review will be published in English.

REVIEW AFFILIATION, FUNDING AND PEER REVIEW

Review team members
Mr Zhiqiang Li. ORCID: 0000-0003-3732-6613. liaocheng people's hospital. China.

No conflict of interest declared.

Mrs shuhui wu (review guarantor). liaocheng third people's hospital. China.

No conflict of interest declared.

Named contact
Mr Zhiqiang Li (lizhiqiangmc@163.com). ORCID: 0000-0003-3732-6613. liaocheng people's hospital. China.

Review affiliation
Liaocheng People's Hospital, Liaocheng, Shandong, China

Funding source
Review has no funding and no agreed support from an academic institution and is done in authors' own time.

Peer review
There has been no peer review of this planned review.

ADDITIONAL INFORMATION

Additional information
This NMA updates and extends Xu et al. (Frontiers in Neurology, 2026; PMID: 41561343 IF: 2.8 Q2). Key differences: (1) lesion specificity — exclusively vertebral artery origin (V1 segment), not mixed intracranial/extracranial; (2) updated evidence — three new studies (Luo 2026 RCT, n=179; Li 2025, n=178; Zhao 2026, n=191) plus Zhang 2020 and Chen 2022 reclassified; (3) analytical depth — dual frequentist and Bayesian NMA with full node-splitting inconsistency diagnostics, leave-one-out sensitivity, and comparison-adjusted

funnel plot. Seven studies (3 RCTs + 4 cohorts, N=931), all from China. PRISMA-NMA compliant. Note: This is a retrospective registration — analysis was completed prior to PROSPERO submission.

Review conflict of interest

Declared individual interests are recorded under team member details.. No additional interests are recorded for this review.

Medical Subject Headings

Adult; Angioplasty; Angioplasty, Balloon; Constriction, Pathologic; Drug-Eluting Stents; Everolimus; Humans; Ischemia; Network Meta-Analysis; Paclitaxel; Stroke; Vertebral Artery

SIMILAR REVIEWS

Check for similar records already in PROSPERO

PROSPERO identified a number of existing PROSPERO records that were similar to this one (last check made on 3 June 2026). These are shown below along with the reasons given by that the review team for the reviews being different and/or proceeding.

- Drug-Coated Balloon Versus Bare-Metal Stent for Neurovascular Stenosis: A Systematic Review and Meta-Analysis of Randomized Controlled Trials [published 14 April 2026] [CRD420261368270]. The review was acknowledged as **similar** but the authors opted to continue because *the review looks at additional or different outcomes*
- Drug-Eluting Stents Vs Bare-Metal Stents for Vertebral Artery Stenosis: A Systematic Review and Meta-analysis [published 5 December 2021] [CRD42021289303]. The review was acknowledged as **similar** but the authors opted to continue because *the review looks at additional or different outcomes*
- Drug-Coated Balloons versus Drug-Eluting Stents for Coronary Artery Disease: A Systematic Review and Meta-Analysis of Randomized Controlled Trials [published 30 March 2026] [CRD420261355942]. The review was judged **not to be similar**

PROSPERO version history

- Version 1.0, published 03 Jun 2026

Disclaimer

The content of this record displays the information provided by the review team. PROSPERO does not peer review registration records or endorse their content.

PROSPERO accepts and posts the information provided in good faith; responsibility for record content rests with the review team. The guarantor for this record has affirmed that the information provided is truthful and that they understand that deliberate provision of inaccurate information may be construed as scientific misconduct.

PROSPERO does not accept any liability for the content provided in this record or for its use. Readers use the information provided in this record at their own risk.

Any enquiries about the record should be referred to the named review contact
