# Supplementary material for: Drug-coated balloon, drug-eluting stent, versus bare-metal stent for vertebral artery ostial stenosis: a systematic review and network meta-analysis
Source: Front Neurol. 2026 Oct 8;17:1901781. doi: 10.3389/fneur.2026.1901781 (PMC13686859; doi:10.3389/fneur.2026.1901781)
Supplement: Supplementary file 2 [file Table_1.docx]

**Supplementary Table S1. Database Search Strategies**

Search date: May 2026. Filter: 2010–2026. No language restrictions.

| Database | SearchStrategy | Records |
| --- | --- | --- |
| PubMed | ("vertebral artery"[MeSH] OR "vertebral artery stenosis"[tiab] OR "vertebral artery origin"[tiab] OR VAOS[tiab]) AND (stent[tiab] OR balloon[tiab] OR angioplasty[tiab] OR DCB[tiab] OR DES[tiab] OR "drug-coated"[tiab] OR "drug-eluting"[tiab]) AND (ostium[tiab] OR origin[tiab] OR "V1"[tiab] OR proximal[tiab] OR extracranial[tiab]) | 387 |
| Embase | Line 1: ‘drug coated balloon’/exp OR ‘drug eluting stent’/exp OR ‘bare metal stent’/exp Line 2: ‘vertebral artery’/exp OR ‘vertebral artery obstruction’/exp Line 3: ostium:ti,ab,kw OR origin:ti,ab,kw OR ‘v1 segment’:ti,ab,kw OR ‘v1’:ti,ab,kw Combined with AND | 35 |
| Cochrane CENTRAL | (“vertebral artery”):ti,ab,kw AND (stent OR balloon OR DCB OR DES OR “drug-coated” OR “drug-eluting”):ti,ab,kw AND (ostium OR origin OR “V1” OR “V1 segment”):ti,ab,kw Filters: Trials only | 19 |
| CNKI | SU = (‘椎动脉’ + ‘椎基底动脉’) * (‘开口’ + ‘起始部’ + ‘V1段’) * (‘支架’ + ‘球囊’ + ‘药物涂层’ + ‘药物洗脱’ + ‘金属裸支架’ + ‘介入’ + ‘血管成形’) | 23 |
| Wanfang | 主题:(“椎动脉” AND (“开口” OR “起始部”)) AND 主题:(“支架” OR “球囊” OR “介入” OR “药物涂层” OR “药物洗脱”) | 35 |
| Total |  | 499 |
| Duplicates removed | EndNote automated deduplication across all databases | 106 |
| Final after deduplication | Included for title/abstract screening | 393 |

DCB, drug-coated balloon; DES, drug-eluting stent; BMS, bare-metal stent; VAOS, vertebral artery origin stenosis.
