# Supplementary material for: Drug-coated balloon, drug-eluting stent, versus bare-metal stent for vertebral artery ostial stenosis: a systematic review and network meta-analysis
Source: Front Neurol. 2026 Oct 8;17:1901781. doi: 10.3389/fneur.2026.1901781 (PMC13686859; doi:10.3389/fneur.2026.1901781)
Supplement: Supplementary file 3 [file Table_2.docx]

Supplementary Table S2. Perioperative Complications (30-Day) by Treatment Arm

| **Study** | **Arm** | **N** | **Stroke** | **TIA** | **Death** | **Total Events** | **Rate** | **Notes** |
| --- | --- | --- | --- | --- | --- | --- | --- | --- |
| Luo 2026 | DCB | 91 | ^a | ^a | 0 | 0 | 0.0% | Composite of TIA/stroke/death = 0/91 |
| Luo 2026 | BMS | 88 | ^a | ^a | 0 | 1 | 1.1% | Composite = 1/88; no deaths |
| Wang 2021 | DCB | 49 | 1^b | 0 | 0 | 2 | 4.1% | 1 cerebral hemorrhage + 2 DWI embolizations^c |
| Wang 2021 | BMS | 46 | 1^b | 0 | 0 | 9 | 19.6% | 1 clinical event + 9 DWI embolizations^c |
| He 2019 | DES | 66 | NR | NR | NR | 3 | 4.5% | Composite periop; no deaths in pilot phase |
| He 2019 | BMS | 66 | NR | NR | NR | 7 | 10.6% | Composite periop |
| Chen 2022 | DCB | 5 | 0 | 0 | 0 | 0 | 0.0% |  |
| Chen 2022 | DES | 29 | 1^d | 0 | 0 | 1 | 3.4% | Perioperative complication in DES group only |
| Chen 2022 | BMS | 12 | 0 | 0 | 0 | 0 | 0.0% |  |
| Zhang 2020 | DES | 55 | NR | NR | NR | NR | NR | Perioperative complications not reported |
| Zhang 2020 | BMS | 55 | NR | NR | NR | NR | NR |  |
| Sun 2025 | DES | 150 | 1^e | 0 | 0 | 1 | 0.7% | Primary safety endpoint: 30-day stroke/death |
| Sun 2025 | BMS | 67 | 0 | 0 | 0 | 0 | 0.0% |  |
| Zhao 2026 | DES | 93 | NR | NR | 0 | 2 | 2.2% | Periprocedural complications: stroke, mortality; no deaths |
| Zhao 2026 | BMS | 98 | NR | NR | 0 | 1 | 1.0% | No deaths occurred in either group |

*^a Luo 2026: Individual TIA vs stroke counts not separately reported; composite 30-day TIA/stroke/death endpoint used.* *^b Wang 2021: 1 clinical perioperative complication per group (cerebral hemorrhage in DCB group).* *^c Wang 2021: DWI-detected asymptomatic embolization: DCB 2/49 (4.1%), BMS 9/46 (19.6%). These are included in periop totals per extraction protocol.* *^d Chen 2022: Nature of perioperative complication not specified beyond being in DES group.* *^e Sun 2025: 30-day stroke rate; death rate was 0%.* *NR = Not reported or not separately extractable from published text.*
