# Supplementary material for: Drug-coated balloon, drug-eluting stent, versus bare-metal stent for vertebral artery ostial stenosis: a systematic review and network meta-analysis
Source: Front Neurol. 2026 Oct 8;17:1901781. doi: 10.3389/fneur.2026.1901781 (PMC13686859; doi:10.3389/fneur.2026.1901781)
Supplement: Supplementary file 4 [file Table_3.docx]

Supplementary Table S3. CINeMA Certainty of Evidence Assessment

| Domain | DCB vs BMS | DES vs BMS | DES vs DCB |
| --- | --- | --- | --- |
| Studies contributing (k) | 3 (1 RCT, 2 Obs) | 5 (2 RCT, 3 Obs) | Indirect (1 three-arm study) |
| Within-study bias | Some concerns^a | Some concerns^a | Some concerns^a |
| Reporting bias | Undetected | Undetected | Undetected |
| Indirectness | No concerns | No concerns | **Major concerns**^b |
| Imprecision | Some concerns^c | No concerns | **Major concerns**^d |
| Heterogeneity | No concerns | No concerns | Not estimable |
| Incoherence | No concerns | No concerns | Not estimable |
| **Overall Certainty** | **Moderate** | **Moderate** | **Low** |

*^a Downgraded due to inclusion of observational (non-randomized) studies with inherent risk of confounding by indication.* *^b Indirect comparison relying on a single small three-arm study (Chen 2022, n=46) with few DCB patients (n=5).* *^c Wide confidence interval for OR: 0.18-0.83, consistent with some uncertainty in effect magnitude.* *^d Very wide confidence interval: OR 1.42, 95% CI 0.58-3.50, crossing the null. Small number of events.*

*CINeMA = Confidence in Network Meta-Analysis (Nikolakopoulou et al., 2020). Domains rated as: No concerns, Some concerns, or Major concerns. Overall certainty: High, Moderate, Low, or Very Low.*
