# Supplementary material for: Drug-coated balloon, drug-eluting stent, versus bare-metal stent for vertebral artery ostial stenosis: a systematic review and network meta-analysis
Source: Front Neurol. 2026 Oct 8;17:1901781. doi: 10.3389/fneur.2026.1901781 (PMC13686859; doi:10.3389/fneur.2026.1901781)
Supplement: Supplementary file 5 [file Table_4.docx]

Supplementary Table S4. Risk of Bias Assessment

| **Study** | **Design** | **Tool** | **D1** | **D2** | **D3** | **D4** | **D5** | **Overall** |
| --- | --- | --- | --- | --- | --- | --- | --- | --- |
| Luo 2026 | RCT | RoB 2 | Low | Low | SC | Low | Low | Some concerns |
| Wang 2021 | RCT | RoB 2 | Low | Low | SC | Low | Low | Some concerns |
| He 2019 | RCT | RoB 2 | Low | Low | Low | Low | Low | Low |
| Chen 2022 | Cohort | NOS | 4/4 | - | - | 1/2 | 3/3 | 8/9 (High) |
| Zhang 2020 | Cohort | NOS | 4/4 | - | - | 1/2 | 3/3 | 8/9 (High) |
| Sun 2025 | Cohort | NOS | 4/4 | - | - | 1/2 | 3/3 | 8/9 (High) |
| Zhao 2026 | Cohort | NOS | 4/4 | - | - | 2/2 | 3/3 | 9/9 (High) |

*RoB 2: D1=Randomization, D2=Deviation, D3=Missing, D4=Measurement, D5=Selection. SC=Some concerns. NOS: max 9; >=7 high.*
