# Supplementary material for: Drug-coated balloon, drug-eluting stent, versus bare-metal stent for vertebral artery ostial stenosis: a systematic review and network meta-analysis
Source: Front Neurol. 2026 Oct 8;17:1901781. doi: 10.3389/fneur.2026.1901781 (PMC13686859; doi:10.3389/fneur.2026.1901781)
Supplement: Supplementary file 6 [file Table_5.docx]

Supplementary Table S5. Devices Used in Included Studies

| **Study** | **Arm** | **Device Name** | **Manufacturer** | **Drug Coating** | **Notes** |
| --- | --- | --- | --- | --- | --- |
| Luo 2026 | DCB | Drug-coated balloon (unnamed in text) | Not reported | Paclitaxel | Bailout stenting with Apollo (MicroPort) in 22% of cases |
| Luo 2026 | BMS | Not specified | Not reported | N/A |  |
| Wang 2021 | DCB | Drug-coated balloon (unnamed in text) | Not reported | Paclitaxel | Used with embolic protection device (EPD) |
| Wang 2021 | BMS | Express | Boston Scientific, Marlborough, MA, USA | N/A |  |
| He 2019 | DES | Maurora (sirolimus-eluting cerebrovascular stent) | Alain Biotechnology Co. Ltd., Beijing, China | Sirolimus | L605 cobalt-chromium alloy; dedicated cerebrovascular DES |
| He 2019 | BMS | Apollo | MicroPort Scientific Corp., Shanghai, China | N/A | 316L stainless steel |
| Chen 2022 | DCB | Orchid (paclitaxel-eluting balloon) | Acotec, Beijing, China | Paclitaxel | Inflated for 60 seconds across stenosis |
| Chen 2022 | DES | XIENCE | Abbott, Temecula, CA, USA | Everolimus |  |
| Chen 2022 | BMS | Apollo | MicroPort, Shanghai, China | N/A |  |
| Zhang 2020 | DES | Xience V / Endeavor / Firebird | Abbott (USA) / Medtronic (Ireland) / MicroPort (China) | Everolimus / Zotarolimus / Sirolimus | Mixed DES types used |
| Zhang 2020 | BMS | Blue / Express | Cordis Corp (Netherlands) / Boston Scientific (USA) | N/A | Mixed BMS types used |
| Sun 2025 | DES | Bridge or Maurora (dedicated VAOS DES) | MicroPort NeuroTech (Shanghai) / Alain Medical (Beijing) | Rapamycin | Balloon-expandable L605 CoCr alloy; up to 4.5-5.0 mm diameter |
| Sun 2025 | BMS | Not specified | Not reported | N/A |  |
| Zhao 2026 | DES | Rapamycin-eluting stent (brand not specified) | Not reported | Rapamycin (sirolimus) | V1 segment-specific |
| Zhao 2026 | BMS | Not specified | Not reported | N/A |  |

*Data extracted from full-text PDFs of each included study. NR = Not reported in manuscript.*
